# Application of a sub-specialties management model improves quality control in a central sterile supply department

**DOI:** 10.1186/s12913-018-3214-7

**Published:** 2018-05-30

**Authors:** Li Wang, Xuejiao Cai, Ping Cheng

**Affiliations:** Central Sterile Supply Department, First Affiliated Hospital of Soochow University, Suzhou City, 215008 Jiangsu Province China

**Keywords:** Sub-specialties, Central sterile supply department, Quality control, Medical device

## Abstract

**Background:**

The management of medical devices is crucial to safe, high-quality surgical care, but has received little attention in the medical literature. This study explored the effect of a sub-specialties management model in the Central Sterile Supply Department (CSSD).

**Methods:**

A traditional routine management model (control) was applied from September 2015 through April 2016, and a newly developed sub-specialties management model (observation) was applied from July 2016 through February 2017. Health personnel from various clinical departments were randomly selected to participate as the control (*n* = 86) and observation (*n* = 90) groups, respectively. The groups were compared for rates of personnel satisfaction, complaints regarding device errors, and damage of medical devices.

**Results:**

The satisfaction score of the observation group (95.8 ± 1.2) was significantly higher than that of the control (90.2 ± 2.3; *P* = 0.000). The rate of complaints of the observation group (3.3%) was significantly lower than that of the control (11.6%; *P* = 0.035). The quality control regarding recycle and packing was significantly higher during the observation period than the control period, which favorably influenced the scores for satisfaction. The rate of damage to specialist medical devices during the observation period (0.40%) was lower than during the control period (0.61%; *P* = 0.003). The theoretical knowledge and practical skills of the CSSD professionals improved after application of the sub-specialties management model.

**Conclusions:**

A management model that considers the requirements of specialist medical devices can improve quality control in the CSSD.

## Background

The central sterile supply department (CSSD) of a health care facility provides sterilized materials to wards, operating rooms, transplant units, and outpatient departments. The CSSD is located in the hospital and is responsible for the recycling, cleaning, sterilization, inspection, packaging, and delivery of all medical devices to various users in the hospital [1]. The reliability of sterile supplies from the CSSD greatly depends on the quality control of the sterilization process. Indeed, good sterilization and packaging practices provided by the CSSD directly influence the quality of health care [2], and more importantly, patients’ safety [2, 3]. Therefore, it is of utmost importance to ensure the quality control of the sterilization process. However, with technological improvements in medical care, the medical devices used in clinics have become more complicated and specialized. Mishandling of these advanced medical devices by the CSSD can lead to damage and poor sterilization. This is considered a challenge in the CSSD.

With time the practice of medicine has become divided into various and refined sub-specialties [4] reflected by clinical departments such as anesthesia [5, 6], pathology [7], and burns [8]. However, there has been no concomitant specialization within the CSSD for providing services to these clinics. The traditional management model of the CSSD is no longer satisfactory, and recent studies of CSSD management methods have shown limited improvements in quality control [9]. Establishing a management model for the CSSD that considers the needs of the sub-specialty clinics would be helpful to adapt services to meet these challenges more appropriately [10]. However, to our best knowledge, no previous study has evaluated the role of a sub-specialties management model as it influences quality control in the CSSD.

The present study investigated the effect of a sub-specialties management model on quality control and health professional’s satisfaction within the CSSD.

## Methods

### Study design and study participants

This is a retrospective study comparing 2 management models in our hospital. A traditional routine management model (described below) was applied in our hospital from September 2015 through April 2016, and this was set as the control treatment. In contrast, the observation management model was applied based on a sub-specialties management model that was implemented from July 2016 through February 2017. A specialist device was defined as a device used by one specific clinical department. The study participants comprised healthcare personnel that were randomly selected from the following departments: operating room, stomatology, gynecology, orthopedics, thoracic surgery, urinary surgery, and general surgery.

A total of 92 staff members were randomly selected to constitute the control group, and 100 staff members were randomly selected for the observation group. Questionnaires were sent to the control group in May 2016 and to the observation group in March 2017. Questionnaires were designed based on relevant studies in the literature with some revisions made by our center [11, 12]. All recruited staff members during the control and observation periods were invited to answer our questionnaire.

The questionnaire was a modified version based on previous work by Shan et al. [13] with good reliability and validity (Cronbach α = 0.978; validity = 0.963). The questionnaire mainly consisted of 3 domains (recycling, packaging, and sterilization) including 21 items, in which participants scored their satisfaction regarding the services provided by the CSSD during the previous 6 months. For the satisfaction survey, participants were asked to reply by selecting one rating from five categories, ranging from ‘Strongly satisfied’ to ‘Strongly dissatisfied’. The answer ‘Strongly satisfied’ was assigned a score of 5 points, while ‘Strongly dissatisfied’ was assigned a score of 1. The satisfaction score was the sum of these scores. The questionnaire also collected data regarding participants’ general information (age, gender, clinical department, highest education level, and professional title). In our hospital, all professionals were classified into 3 groups according to their professional level: low, medium, or high.

We also conducted tests of knowledge and skills among the professional staff (*n* = 27) on March 2016 and January 2017 during the control and observations periods, respectively, in the CSSD.

### Management models

#### Control treatment

For the control treatment (routine management), the CSSD consisted of 3 working zones: cleaning; sterilization and packaging; and sterile storage. A team leader was assigned to each zone, who assisted the head supervisor of the CSSD to complete all the work of the zone. The working professionals in the CSSD were randomly assigned to the different working zones.

#### Observation treatment

The observation treatment (i.e., sub-specialty management model) supplemented the traditional model with various modules to improve quality control. Based on the results of a medical device-related scale evaluation, in the CSSD specialist medical devices were classified into 3 sub-specialties: laparoscopic; stomatology; or rental. Staff personnel in the CSSD were assigned to the different sub-specialty groups based on their professional title, working years in the CSSD, highest educational level, working ability, and personal willingness.

Each sub-specialty group took full responsibility for the cleaning, disinfection, sterilization, and packaging of medical devices within that sub-specialty. The CSSD professionals in each group worked closely with the relevant clinical departments. Clinicians in the relevant departments were regularly invited to deliver speeches on the clinical use and maintenance of specific medical devices. All the sub-specialty group members worked in the cleaning zone, and the sterilization and packaging zone. Shifts between different sub-specialty groups took place every 6–12 months.

### Statistical analysis

Sample size was calculated based on α = 5% and β = 90%. An increase of ≥10 points of satisfaction score was defined as clinical improvement; the expected sample was ≥81. In consideration of a possible 10% non-response rate, we determined that an invitation letter should be sent to ≥90 participants.

The t-test was used to compare age, satisfaction scores, complaint rate, device damage rate, skill scores, and error rate. The chi-squared test (χ2) was applied to compare education and professional levels. A 2-sided *P*-value < 0.05 was considered statistically significant. All analyses were conducted using the SPSS 17.0 software (SPSS, Chicago, IL, USA).

## Results

We received 86 questionnaires (response rate 93.5%) during the control period, and 90 questionnaires (response rate 90%) during the observation period. The distributions of age, gender ratio, education level, and professional title were similar between the 2 groups (Table 1). The average age of the control and observation groups was 35.9 ± 7.8 years and 37.2 ± 9.0 years, respectively.

**Table 1:** Baseline characteristics of the study population

		Control	Observation	Test value	*P*
Subjects, n		86	90		
Age, y		35.9 ± 7.8	37.2 ± 9.0	*t* = 1.049	0.296
Gender	Male	40 (46.5%)	38 (42.2%)	χ2 = 0.328	0.567
	Female	46	52		
Education level	Associate’s	19	22	χ2 = 0.327	0.849
	Bachelor’s	30	33		
	Master’s or above	37	35		
Professional title	Low	13	14	χ2 = 0.152	0.927
	Medium	33	32		
	High	40	44		

Compared with the control group, the observation group had significantly lower complaint scores (Table 2). The overall satisfaction score of the observation group (95.8 ± 1.2) was significantly higher than that of the control group (90.2 ± 2.3; *P* = 0.000). The quality of recycled devices reported by the observation group (32.8 ± 4.5) was significantly higher than that of the control group (30.2 ± 4.2; *P* = 0.000). Furthermore, the quality of device packing reported by the observation group (32.4 ± 4.2) was significantly higher than that of the control group (30.9 ± 3.6; *P* = 0.01). The sterilization quality of devices reported by the 2 groups was comparable (*P* = 0.131).

**Table 2:** Degree of satisfaction and complaint rate between control and observation groups

		Control	Observation	Test value	*P*
Subjects, n		86	90		
Satisfaction scores		90.2 ± 2.3	95.8 ± 1.2	*t* = 19.80	0.000
	Recycle quality	30.2 ± 4.2	32.8 ± 4.5	*t* = 4.00	0.000
	Packing quality	30.9 ± 3.6	32.4 ± 4.2	*t* = 2.59	0.010
	Sterilization quality	29.2 ± 6.1	30.6 ± 6.3	*t* = 1.52	0.131
Complaint rate		11.6%	3.3%	χ2 = 4.42	0.035

In the control group, 44,652 pieces of medical devices were cleaned and sterilized. Of these, 21,780 pieces were specialist medical devices, among which 132 (0.61%) were damaged in the CSSD (Table 3). During the observation period, 50,445 pieces of medical devices were cleaned and sterilized. Of these, 22,044 were specialist medical devices, among which 89 (0.40%) were damaged in the CSSD. Thus, the damage rate of specialist medical devices during the observation period (0.40%) was significantly lower in that of the control period (0.61%; *P* = 0.003).

**Table 3:** Evaluation of CSSD professionals’ theoretical knowledge, and practical skills, and error rate during work

	Control	Observation	Test value	*P*-value
Theoretical knowledge	87.3 ± 5.5	93.7 ± 5.0	*t* = 3.40	0.002
Practical skill	86.4 ± 4.7	91.6 ± 5.1	*t* = 2.88	0.009
Error rate	0.92% (409/44652)	0.52% (263/50445)	χ2 = 52.57	0.000
Device damage	0.61% (132/21780)	0.40% (89/22044)	χ2 = 8.94	0.003

Significant improvement was also observed during the observation period regarding the professional skills of the CSSD workers, of which both theoretical knowledge and practical skills were evaluated. The theoretical knowledge score of the observation group (93.7 ± 5.0) was significantly higher than that of the control (87.3 ± 5.5; *P* = 0.002; Table 3). The scores for practical skills of the CSSD professionals during the observation period (91.6 ± 5.1) were significantly higher than during the control period (86.4 ± 4.7; *P* = 0.009). The medical device damage rate was significantly higher in the control group than the observation group.

## Discussion

The current study explored the application of a sub-specialties management model that can ensure good sterilization practices in the CSSD. We developed sub-specialty modules that were based on clinical need, with specified management for various medical devices. Our study indicates that the application of a management model that considered sub-specialties in the CSSD increased the satisfaction of the clinical healthcare staff, decreased the number of complaints, and lowered rates of medical device damage and error. These improvements are partly due to the enhanced theoretical knowledge and practical skills of the CSSD professionals.

Previous studies have also explored potential methods to improve control of safety in the CSSD. Debabrata et al. [10] reported that retaining sterilization records was helpful to ensure high safety control, as recordkeeping facilitated monitoring the quality of daily work practices. In China and some other countries, hospitals have increasingly applied an instrument tracking system in the CSSD, which has reduced the risk of errors in packaging surgical instruments, including missing and mismatched instruments [14, 15].

The physical infrastructure of the CSSD consists of separate work areas for decontamination, packaging, linen preparation, and sterile storage. Clear job assignments are highly important in the CSSD layout of these areas. In the present study, the designed management model specified a job assignment with experienced personnel for each working module. This subsequently reduced the error rate of the entire process.

A previous study identified job assignment as an important indicator of the performance of CSSD quality control [16]. The study also suggested that CSSD personnel require continuing professional development through education, otherwise they will not be able keep up with advancements in the technology of new machines. Continuing professional development is a component of our management model, whereby CSSD professionals receive knowledge and training regarding the clinical use of medical devices. In the present study, the training program designed for the packaging staff in the CSSD was shown to be helpful for improving their knowledge of equipment. Staying educated in the CSSD is not easy, but is necessary for providing effective and efficient service [17].

The CSSD is often a neglected area of infection control in hospitals. Investment in a well-equipped CSSD infrastructure is essential for the smooth functioning of a hospital. Resources should be directed not only for the development of the physical infrastructure and equipment in the CSSD, but also for the recruitment and retention of technically qualified personnel who are able to operate the system effectively [18].

In the present study, it should be noted that the clinics’ satisfaction score for sterilization remained unchanged after application of the sub-specialty model. Quality control of the sterilization process is complex. A previous study suggested that quality assurance for sterilization can depend on several factors, including compliance with sterilization guidelines, validation of sterilization instruments, the use of chemical and biological indicators, and precautions against sharp injury [16]. The Association for the Advancement of Medical Instrumentation and the International Standards Organization has provided recommendations regarding physical parameters and chemical and biological indicators that ensure sterilization quality control. Debabrata et al. [19] shared their experience with the application of various sterilization indicators in the CSSD of a 167-bed oncology hospital in eastern India.

Errors in instrument processing can increase operative time and costs, and can potentially contribute to surgical infections and perioperative mortality [20, 21].

Our present study found that the rate of reported errors was significantly lower during the observation period than during the control period. It has been reported that 44.0% of errors in a Chinese hospital occurred because of errors in the labeling of packed instruments, and mainly among instruments of similar type (currently in manuscript). Instruments of similar structure are frequently mixed, and this is usually due to personnel error. Hospital personnel working in the CSSD are unfamiliar with the application of surgical instruments in clinics, which increases their difficulties in distinguishing between instruments with minor differences. In the present study, the continuous training provided by our management model successfully increased the knowledge and skills of the CSSD professionals. This was probably responsible for the subsequent reduction in errors during the sterilization or packaging processes.

The error rate may furthermore be reduced by using the Lean method [22], which improves the processing of surgical instruments by redefining operator roles, altering the workplace, mistake-proofing, quality monitoring, staff training, and continuous feedback. The Virginia Mason Medical Center in Seattle, WA, improved the process of surgical instrument sterilization by applying Lean production improvement methods, and entitled this the Virginia Mason Production System [22]. Instrument processing errors decreased from 3.0 to 1.5%, in particular assembly errors in packaging (from 0.66 to 0.24 errors per 100 cases).

## Conclusion

The current study demonstrated the beneficial effects of instituting the sub-specialty management model for quality control of medical devices in the CSSD.

## Abbreviations

AAMI: Advancement of Medical Instrumentation
CSSD: Central Sterile Supply Department
ISO: International Standards Organization
VMPS: Virginia Mason Production System
